# Focusing on Transgender Healthcare

**DOI:** 10.1089/trgh.2015.29001.edi

**Published:** 2016-01-01

**Authors:** Robert Garofalo

**Affiliations:** Northwestern University; Ann & Robert H. Lurie Children's Hospital, Chicago, Illinois.

In life, the paths we choose are influenced by our day-to-day experiences and the people we meet. Career choices are no different. As a pediatrician and academic, I am not infrequently asked how I became interested in working with the transgender community and doing academic work with this population. An openly gay man, many wrongfully assume that my interest and devotion stem from an identity as a sexual minority, but let us be honest that the lesbian, gay, and bisexual community has not always stood (as they should) in support and solidarity for transgender people, so quite honestly, my identity as a gay man has not been at the core of my commitment to transgender people or my interest in transgender health. My typical answer to that question credits my parents and my upbringing. My parents were middle school and high school educators teaching in inner city public high schools. They led by example and so rather naturally I developed an interest in caring for the underserved.

For far too long, transgender people and transgender health have been footnotes in the fields of academic medicine and public health. Healthcare is a fundamental right of every human being; however, transgender people have often been overlooked in their pursuit of the highest quality healthcare, in part, because they face significant barriers when it comes to basic needs, including social support, education, housing, employment, and access to medical and mental health services. Among the many disparities facing transgender people are extreme levels of violence and harassment, and experiences of discrimination in the healthcare environment. Transgender people, particularly transgender women from communities of color, are the highest risk demographic group in the United States for the acquisition of HIV. Transgender people are also at increased risk for mental health and substance use issues, including higher risk for depression, post-traumatic stress, and attempted suicide. However, transgender health and the disparities-affected transgender people are woefully under-represented in the peer-reviewed academic literature. And as a good friend of mine once said, “in order to count, you need to be counted.”

As I assume the role of Editor-in-Chief of *Transgender Health*, it is the intent to both shine a light through academic work on the already mentioned disparities and, more importantly, to be part of steps to help eliminate them. Assuming this role is a distinct honor for me and has given me the opportunity to reflect back on the roots of my personal interest in transgender health. Wedded in caring for the underserved as many of us who do this work, it has been four seminal life experiences that led me down the path of academic and clinical work caring for children and adolescents who are gender nonconforming or who identify as transgender.

In 1990, I met Marsha P. Johnson, the gay liberation and AIDS activist, at a community event in New York City while attending medical school. I love pointing out to people that the “P” was her trademark and it stood for “pay it no mind” as I think that positive spirit of defiance is something I have always admired and respected and something that over the years has served the transgender civil rights and health movement well. To my knowledge, Ms. Johnson was the first transgender person I had ever met. To put it rather bluntly, I was mesmerized by her. I was a meek, nerdy cis-gender white man and she was this tall, powerful black drag queen. She talked about her life's work and activism helping young drag queens and other homeless youth get food and clothing. I sat there in awe. Marsha and I were never friends; in fact we never met again. But I never forgot her. Shortly after graduation I read in horror and disbelief about her death in 1992; she was found floating in the Hudson River near the Greenwich Village piers where she once worked to help others. I thought back to that moment we met, how I sat in awe of her presence and her commitment to the community. I knew then that transgender work or engagement with the transgender community would be part of my life in the future.

In 1995, I moved to Boston after finishing a pediatric residency at the Children's Hospital of Philadelphia for a clinical fellowship in pediatric advocacy. I had the privilege of working at the Sidney Borum Health Center, a new and innovative care model for homeless and disenfranchised youth. In my first weeks, Bob Garcia the Director of Social Work brought me a 16-year-old young transgender woman. Her chief complaint was, “I want to start hormones.” I had never heard that before and remember panicking a bit. “Are you kidding me?” I learned nothing about cross-sex hormones in medical school or residency. None of us did back then. I remember asking, “Is it ethical to give a 16 year old boy estrogen?” I had a lot to learn in my own understanding of gender. Bob took a deep breath and said, “I understand this is new to you. But get to know her. She has lived her life as a girl since the age 7. She sees a mental health provider weekly. The pertinent question isn't, is it ethical to give her estrogen. The correct question is, is it ethical not to.” So, get to know her I did. And with self-taught education and mentorship from staff at the Borum, my mindset shifted and my first prescription for cross-sex hormones was written. The Borum fundamentally changed my aspirations for a clinical and academic career. I saw the need for education and training about transgender health in healthcare professional curriculum and experiential learning opportunities. I saw through the lens of my patients the harsh social realities facing transgender people and the challenges, in particular, facing transgender women with regard to homelessness, violence, and HIV. I cared for my first HIV-infected primary care adolescent patient at the Borum; it was a young transgender woman.

In 2010, I flew to New York City to participate in a *Dr. Oz Show* segment on transgender kids. I was not their first choice for the segment (Norman Spack, MD, from Harvard was). I was out of my comfort zone as my career had focused exclusively on transgender adolescents, mostly young transgender women and HIV clinical care and prevention. There I was in the NBC Studios, face-to-face with Dr. Oz and his panel of guests, including some very young transgender children answering questions about the health and development of transgender children. I remember making the point as the “medical expert” that as pediatricians our goal for the healthcare of gender nonconforming children is no different than for other populations of young people and that is to focus on creating a healthy, nurturing, and supportive environment for children to thrive. Following the episode, families with gender nonconforming children as young as 4 or 5 years of age suddenly began to use the Internet to contact me to come in for healthcare. Children and families from as far away as Arkansas and Kansas (I am in Chicago) were looking for a medical provider to help provide education, guidance, and care. This experience combined with the mentorship and encouragement of colleagues and friends such as Johanna Olson, MD, and Stephen Rosenthal, MD, led to a change (some might say addition to) in my career path to think more broadly about the transgender health and specifically to the inclusion of children and families.

In 2011, the National Academies of Sciences and the Institute of Medicine (IOM) published a committee report on The Health of Lesbian, Gay, Bisexual, and Transgender People. This report was commissioned by the National Institutes of Health (NIH) to “assess the current state of knowledge about the health of lesbian, gay, bisexual and transgender (LGBT) people, as well as to identify research gaps and to formulate an academic research agenda that could guide the NIH in enhancing and focusing its research in this area.” This was a watershed moment in the history of transgender health. It was an honor to serve on this committee along with 16 of my colleagues in the field of LGBT health. One of the primary focuses of this committee's work and recommendations as it relates to transgender people was to specifically use a life course framework and intersectional perspective that recognized that sexual and gender-minority status is only one of many factors that influence the lives and health of transgender individuals. For me personally, it helped continue to broaden the lens from which I viewed the health and well-being of transgender people beyond the narrow focus of HIV to being more holistic and inclusive of a range of medical and healthcare issues ranging from primary care to behavioral health to surgical interventions and outcomes.

Consistent with the goals of the IOM*, Transgender Health* establishes a timely international journal that focuses on the publication of peer-reviewed articles dedicated to better understanding the healthcare needs of transgender people throughout their lifespan. It is our hope that *Transgender Health* becomes for some one of those “life experiences”—like mine already mentioned—that motivates people to become more involved in doing academic work and engaging with the transgender community by creating a space for a growing body of literature that is diverse in its scope and moves us forward toward a model of healthcare equity and cultural humility for this underserved and often misunderstood community. We are committed to addressing important gaps in the health disparities literature and focusing on much-needed interventions to improve the lives of transgender people. We will focus on priority areas where policy development and research are needed to better healthcare (e.g., medical, surgical, and behavioral) outcomes. As an open access journal, *Transgender Health* seeks to build an open network to share clinical knowledge and skills so that models of care can be developed to best meet the needs of the community and so that healthcare providers can deliver the highest quality care to transgender people. To that end, I want to thank Mary Ann Liebert personally for her dedication to this community. Mary Ann has been a leader in the field of LGBT health for decades and her personal commitment to *Transgender Health* was evident to me from the moment she called to talk about me becoming Editor-in-Chief. I also want to thank the dedicated and talented staff at Mary Ann Liebert, Inc., Publishers, most notably Jordan Schilling, the managing editor of *Transgender Health.* With Jordan's help, expertise, and mentorship, we have assembled a talented team here, including an international and diverse Editorial Board led by our Associate Editors, Johanna Olson, MD, Sari Reisner, PhD, Asa Radix, MD, Stephen Rosenthal, MD, and Jae Sevelius, PhD. These individuals represent the very best in this field as well as the next generation of both clinicians and academicians who lead this work well into the future.

We hope that *Transgender Health* will inspire all medical and healthcare professionals, students, and trainees to recognize the urgent need to deliver optimal healthcare to the transgender community and move the needle forward in improving transgender healthcare.

We look forward to your contribution to the Journal.

## Abbreviations Used

IOM: Institute of Medicine
LGBT: lesbian, gay, bisexual, and transgender
NIH: National Institutes of Health

